# Analysis of the Structure and Functioning of the Chudao Oyster Reef Ecosystem in Sanggou Bay, China

**DOI:** 10.1002/ece3.73523

**Published:** 2026-04-20

**Authors:** Yazhou Shi, Qisheng Tang, Yaping Gao, Ruihuan Li, Weiwei Li, Mingjun Yuan, Linjie Wang, Shujie Chang, Zengjie Jiang

**Affiliations:** ^1^ College of the Environment and Ecology Xiamen University Xiamen China; ^2^ State Key Laboratory of Mariculture Biobreeding and Sustainable Goods, Key Laboratory of Carbon Sink Fisheries, Yellow Sea Fisheries Research Institute Chinese Academy of Fishery Sciences Qingdao China

**Keywords:** biodiversity, Ecopath, oyster reef, Sanggou Bay, structure and functioning

## Abstract

The Chudao oyster reef in Sanggou Bay, China represents a rare case of reef expansion in the context of global oyster reef degradation. However, its ecosystem structure and functioning remain poorly understood. To fill this knowledge gap, a whole‐biota survey was conducted in May 2024, covering plankton, microorganisms, reef‐dwelling animals, and nekton across different tidal zones. An Ecopath model was subsequently constructed to quantify trophic structure and energy flow. Results revealed a total of 30 reef‐dwelling animal species, with oysters (*Magallana gigas*) dominant in both density (1335–1605 ind m^−2^) and biomass (12.87–14.04 kg m^−2^). Biodiversity indices did not differ significantly among tidal zones (*p* > 0.05), although relatively higher values were observed in the low‐tide zone. The Ecopath model comprised 17 functional groups with trophic levels ranging from 1.00 to 3.73. Total system throughput (TST) reached 36,752.52 t km^−2^ year^−1^, with primary producers contributing 61% of total energy flow. Ecosystem indices indicated a relatively mature and stable ecosystem, while the overall energy transfer efficiency was low (4.21%). As the first ecosystem‐level assessment of the Chudao Oyster Reef Ecosystem (*CORE*), this study provides valuable quantitative insights into its structural and functional characteristics and establishes a scientific basis for the conservation, management, and sustainable development of this rare expanding oyster reef ecosystem.

## Graphical abstract

The study focuses on the Chudao oyster reef ecosystem in Sanggou Bay, China. By investigating the biodiversity, food web structure, and energy flow, the study aims to provide a basis for further exploring the role of oyster reef in maintaining marine ecological environment.

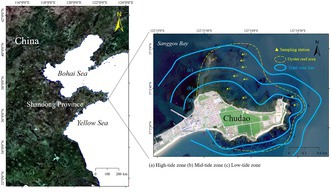

## 1 Introduction

Oyster reefs are widely recognized as ecosystem engineers that provide multiple ecological functions, including water purification (Cornwell et al. 2016; Dame et al. 1984, 1989), habitat provision (McLeod et al. 2020; Humphries et al. 2016; Zu Ermgassen et al. 2016), carbon sequestration (Veenstra et al. 2021; Lee et al. 2020; Fodrie et al. 2017), and shoreline stabilization (Coen et al. 2007). Despite their ecological importance, more than 85% of oyster reefs have been lost or degraded globally over the past century, making them among the most threatened coastal ecosystems (Beck et al. 2011). In contrast to this global decline, extensive oyster reefs have developed and expanded in the Chudao area of Sanggou Bay, northern China. Driven by marine ranching practices and large‐scale aquaculture activities, the Chudao Oyster Reef Ecosystem (hereafter referred to as *CORE*) represents a rare case of reef persistence and growth under strong anthropogenic influence. This ecosystem may play an important role in supporting local biodiversity, supporting trophic interactions, and regulating coastal ecosystem processes. However, its ecological structure and functioning remain poorly understood.

Understanding ecosystem composition across multiple trophic levels is fundamental for assessing ecosystem structure and functioning. Previous studies on oyster reefs have primarily focused on macrofauna, reef‐associated animals, and fish communities (Zu Ermgassen et al. 2016; Shervette and Gelwick 2008; Boudreaux et al. 2006; Tolley and Volety 2005), while other important components, particularly microorganisms, have received considerably less attention. Microbial communities are key drivers of biogeochemical cycling and organic matter transformation, and their omission may lead to incomplete assessments of ecosystem structure and functioning (Hurst et al. 2022; Chambers et al. 2018; Feinman et al. 2018; Green et al. 2012). Therefore, a whole‐biota approach that integrates plankton, microorganisms, benthic organisms, and nekton is necessary to comprehensively characterize oyster reef ecosystems. In addition to biological composition, spatial variation in biodiversity is an important aspect of ecosystem structure, particularly in intertidal ecosystems (Davidson et al. 2004). Oyster reefs are subject to strong environmental gradients across tidal zones, including variations in immersion time, hydrodynamics, and substrate exposure. These gradients may influence species distribution, community composition, and diversity patterns. However, spatial biodiversity patterns in oyster reef ecosystems, especially across tidal zones, remain insufficiently quantified.

Beyond species composition and spatial patterns, ecosystem functioning is governed by trophic interactions and energy flow. Oyster reefs support complex food webs characterized by both grazing and detrital pathways, linking primary producers, benthic consumers, and higher trophic levels. Ecopath, a mass‐balance modeling approach, provides an effective framework for quantifying trophic structure and energy flow in aquatic ecosystems (Pauly 2000; Polovina 1984). Although widely applied in marine ecosystem studies (Capitani et al. 2022; Gao et al. 2020; Colléter et al. 2015), its application in oyster reef ecosystems remains limited (Zhang et al. 2022; Xu et al. 2019), particularly in ecosystems integrating multiple biological components.

To address these knowledge gaps, this study aimed to provide a comprehensive assessment of *CORE* by integrating multi‐trophic biological surveys with ecosystem modeling. Specifically, this study addresses four key questions: (1) What is the ecosystem composition across multiple trophic levels? (2) How does biodiversity vary across tidal zones? (3) What are the main trophic pathways and food web structure? and (4) How is energy transferred within the ecosystem? To answer these questions, we conducted a whole‐biota survey in *CORE* in May 2024, including plankton, microorganisms, reef‐dwelling animals, and nekton, and constructed an Ecopath model to quantify trophic structure and energy flow. This study provides a comprehensive ecosystem‐level assessment of *CORE* and contributes to understanding the functioning of oyster reef ecosystems in coastal environments.

## 2 Materials and Methods

### 2.1 Study Area

**FIGURE 1:**
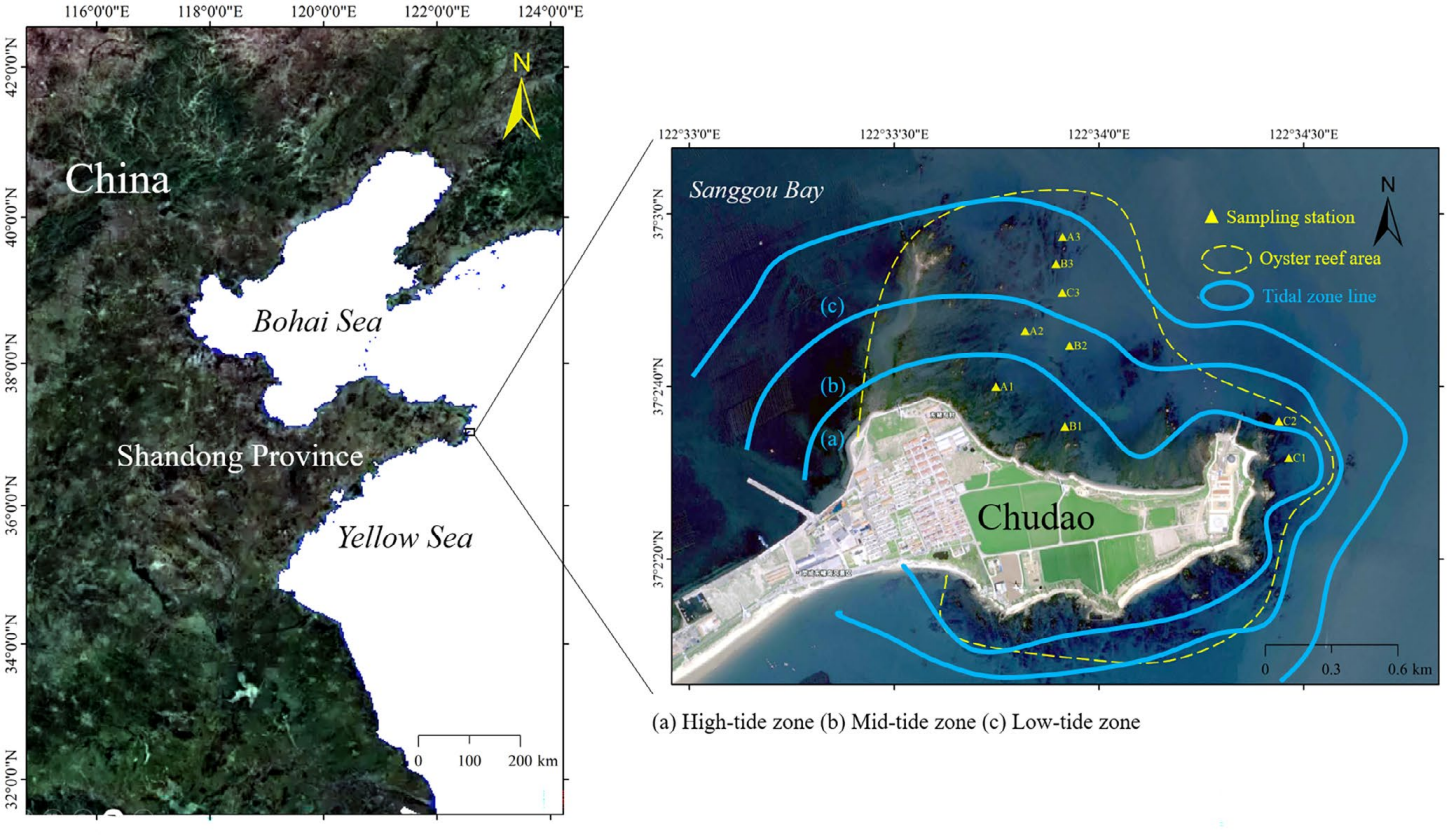
Location of the study area and distribution of sampling stations in *CORE*.

Chudao (37°02′38.3′′ N, 122°33′30.3′′ E) is located in the southern section of the entrance to Sanggou Bay, which lies at the easternmost tip of the Shandong Peninsula (Xing et al. 2023; Zhou et al. 2023). The dominant tidal pattern here is the irregular semi‐diurnal tide (Zeng et al. 2015). To delineate the distribution of oyster reefs in Chudao, we carried out drone‐based aerial surveys in October 2021. The results revealed that these oyster reefs are predominantly concentrated along the northern coastline of Chudao, spanning an area of approximately 0.29 km^2^. For the purpose of this study, nine sampling sites were set up across three transects (A, B, and C) within *CORE*. Each transect included three sites positioned in the high, middle, and low tidal zones respectively (Figure 1).

The field survey was conducted during the low‐tide period, when the tide ranged from −0.2 to −0.1 m, spanning five consecutive days from May 23 to 27, 2024. The environmental parameters measured during the survey are summarized in Table 1. Water temperature (T), salinity (S), dissolved oxygen (DO), and pH were measured in situ using a Manta portable water quality analyzer. Chlorophyll a (Chla) concentrations were determined with a Trilogy fluorimeter (Stelmakh and Alatartseva 2024). Suspended particulate matter (SPM) and particulate organic matter (POM) were quantified via the gravimetric method: samples were filtered through GF/F membranes that had been pre‐calcined at 450°C for 6 h (Chaves et al. 2021).

**TABLE 1:** Environmental parameters (±SD) in *CORE*.

Parameter	High‐tide zone	Mid‐tide zone	Low‐tide zone
*T* (°C)	17.43 ± 0.25	17.31 ± 0.13	18.03 ± 0.90
*S*	31.95 ± 0.09	32.00 ± 0.05	31.82 ± 0.34
DO (mg L^−1^)	7.97 ± 0.17	8.00 ± 0.06	7.95 ± 0.57
pH	8.79 ± 0.08	8.92 ± 0.09	8.74 ± 0.10
Chla (μg L^−1^)	0.78 ± 0.49	0.55 ± 0.14	0.67 ± 0.17
SPM (g L^−1^)	0.06 ± 0.01	0.05 ± 0.01	0.05 ± 0.00
POM (g L^−1^)	0.01 ± 0.00	0.01 ± 0.00	0.01 ± 0.00

### 2.2 Sampling and Analysis Methods

The geographic coordinates (latitude and longitude) of each sampling station were recorded using a GPS device. Multiple biological components spanning different trophic levels were collected in *CORE*, including plankton and microorganisms at the lower trophic levels, reef‐dwelling animals at the intermediate trophic levels, and nekton at the higher trophic levels, reflecting a relatively complete structure of the coastal ecosystem.

For phytoplankton, 500 mL of surface seawater was collected at each station and filtered through a 200 μm plankton net. The samples were then transferred into 500 mL polyethylene bottles and preserved with Lugol's solution. For zooplankton, 5 L of seawater was collected and filtered through a 160 μm plankton net. Retained organisms were rinsed into 500‐mL polyethylene bottles and preserved in 75% ethanol. Plankton samples from three sites along each transect were pooled separately and identified under a microscope based on their morphological characteristics (Manikandan et al. 2024; Costello et al. 2013; The State Bureau of Quality and Technical Supervision 2008; WoRMS Editorial Board n.d.; Guiry and Guiry, n.d.). For microorganisms, a 25 × 25 cm quadrat was randomly placed on the reef sediment at each station. Sampling equipment was sterilized with alcohol swabs before use. Sediment from 4 to 5 points within each quadrat was mixed and transferred into a 5 mL cryotube, immediately placed on dry ice and subsequently stored at −80°C until analysis. Microbial community composition, abundance, and diversity were analyzed using 16S rDNA gene sequencing (Macrae 2000). The sequencing workflow included DNA extraction, PCR amplification, quantitative fluorescence analysis, paired‐end library construction, and Illumina sequencing. Bioinformatics analysis was subsequently performed to obtain amplicon sequence variants (ASVs) and their abundance, followed by taxonomic classification, community diversity analysis, differential species analysis, and functional prediction.

For reef‐dwelling animals, three 25 × 25 cm quadrats were randomly placed on the reef structures at each station. All animals within each quadrat, including oysters, were collected in sealed bags and transported to the laboratory. In the laboratory, organisms were cleaned to remove surface biofouling, preserved in 75% ethanol in 250 mL polyethylene bottles, and subsequently identified. Species composition, abundance, and biomass were determined using a biological identification guide (Leng et al. 2017). For oysters, shell height (SH), abundance, biomass, and recruitment (juveniles with SH < 20 mm; CAOE 2020) were measured using an analytical balance and vernier calipers. In addition, three live oysters were randomly selected from each sampling site (*n* = 27), and muscle tissue was excised from each individual and stored in cryovials at −80°C. Species identification was subsequently conducted using single‐nucleotide polymorphism (SNP) analysis.

For nekton, two transects were randomly selected, and one station from each transect was chosen for the deployment of bottom cages (8 × 0.3 × 0.18 m). The cages were deployed for 24–48 h. The captured nekton specimens were collected, placed in sealed bags, and transported to the laboratory for species identification and abundance analysis.

### 2.3 Dominance and Biodiversity Indices

Species dominance and diversity indices were employed to characterize the community traits of different biota within *CORE*. First, the formula for the species dominance index is as follows:

McNaughton species dominance index (*Y*) (Pinkas et al. 1970):

$$Y=\frac{n_{i}}{N}\times f_{i}$$

where *n*_*i*_ is the total number of individuals of the *i*th species, *N* is the total number of individuals at all stations, *f*_*i*_ is the frequency of occurrence of the species at each station. Usually, species with *Y* > 0.02 are defined as dominant.

Second, species diversity indices include the Shannon‐Wiener index (*H*′), the Margalef species richness index (*d*), and the Pielou species evenness index (*J*′). Their formulas are as follows:

Shannon‐Wiener index (*H*′) (Shannon and Weaver 1949):

$$H^{\prime }=-\sum P_{i}\ln P_{i}$$

Margalef species richness index (*d*) (Margalef 1968):

$$d=\left(S-1\right)/\ln N$$

Pielou species evenness index (*J*′) (Pielou 1975):

$$J^{\prime }=H^{\prime }/\ln S$$

where *N* is the total number of individuals in the community, *n*_*i*_ is the total number of individuals of the *i*th species, *S* is the total number of species, and P_i_ is the ratio of the number of individuals of the *i*th species to the total number of individuals in the community.

### 2.4 Ecopath Model Construction and Analysis

#### 2.4.1 Fundamental Principles

A mass‐balanced trophic model was constructed using the Ecopath with Ecosim (EwE) 6.5. The model includes a set of linear equations that describe mass balance over a given time, which can be expressed as follows:

$$B_{i}\times {\left(\frac{P}{B}\right)}_{i}\times {\mathrm{EE}}_{i}=\sum_{j=1}^{j}B_{j}\times {\left(\frac{Q}{B}\right)}_{j}\times {\mathrm{DC}}_{\mathrm{ij}}+Y_{i}+{\mathrm{BA}}_{i}+E_{i}$$

where *B*_*i*_ and *B*_*j*_ represent the biomass of groups *i* and *j*, respectively; (P/B)_*i*_ is the production‐to‐biomass ratio of group *i*, which is equivalent to the instantaneous total mortality rate; (Q/B)_*j*_ is the consumption‐to‐biomass ratio of predator group *j*; EE_*i*_ denotes the ecotrophic efficiency, representing the proportion of production of group *i* that is utilized within the ecosystem; DC_*ij*_ is the fraction of prey *i* in the diet of predator *j*; *Y*_*i*_ is the fishery yield of group *i*; BA_*i*_ and *E*_*i*_ represent the biomass accumulation rate and net migration rate, respectively.

#### 2.4.2 Functional Groups and Parameters

Species were grouped based on their feeding habits, habitat preferences, and ecological or taxonomic similarities. Species of particular economic or ecological importance in *CORE* were treated as independent functional groups. Ultimately, the ecosystem was divided into 17 functional groups, with each fish species assigned to a separate group. For each functional group, DC_*ij*_ and at least three of the following parameters must be specified: *B*, P/B, EE, and Q/B, whereas the remaining parameter can be estimated by the model (Han et al. 2017).

In this study, Ecopath was applied using wet weight (t km^−2^) to represent energy flow, with a simulation period of one year. All biomass data were derived from the present biological survey. The initial biomass of fish and cephalopods was estimated using the swept‐area method (Han et al. 2017). Detritus biomass was obtained from previous studies conducted in waters adjacent to Sanggou Bay (Wu et al. 2016). Due to the lack of survey data for other demersal fish and benthic algae, the ecotrophic efficiency (EE) was set to a default value of 0.95 for estimation (Han et al. 2017). The P/B, Q/B, and diet composition (DC) parameters for all functional groups were obtained from Ecopath models of adjacent or comparable ecosystems (Liu 2024; Gao et al. 2022; Yuan et al. 2022; Zhang et al. 2022; Lin et al. 2022; Su et al. 2022; Cui and Zhu 2020; Gao et al. 2020; Xu et al. 2019; Lee and Zhang 2018; Ma et al. 2018; Han et al. 2017; Wu et al. 2016). Additionally, fishing mortality (*F*) was set to zero for all functional groups, as *CORE* is located within legally protected areas where fishing activities are strictly prohibited (Li et al. 2025; Bohorquez et al. 2021; Zheng et al. 2013).

To evaluate the impact of the “zero fishing mortality” assumption, a sensitivity analysis was conducted by introducing hypothetical low fishing mortality rates (*F* = 0.05 and *F* = 0.1) for the primary consumer group (i.e., high‐trophic‐level fish). Subsequently, changes in key ecosystem indicators, including trophic transfer efficiency, total system throughput, and Finn's cycling index, were compared against those of the baseline model.

#### 2.4.3 Model Calibration

After inputting the basic data, the model was balanced to ensure equilibrium between ecosystem inputs and outputs, with the constraint that 0<EE≤1. When EE>1 occurred, the input parameters of the affected functional groups were adjusted within ±10% until a balanced state was achieved. Most P/Q ratios after model balancing ranged between 0.1 and 0.3.

After model balancing, ecological analyses in EwE were conducted to examine the trophic structure and functioning of the ecosystem. Primary producers and detritus were assigned a trophic level (TL) of 1, while the TLs of consumers were calculated as 1 plus the weighted average of their prey's TLs. Multiple indicators were then used to characterize ecosystem structure: (1) Trophic interactions among functional groups were quantified using ecotrophic efficiency (EE). (2) The food web structure describes feeding relationships among organisms, forming a complex network that reflects ecosystem complexity. Energy flow diagrams were used to illustrate the efficiency of energy transfer among trophic levels, thereby revealing ecosystem energy balance and the roles of different biological groups. (3) The mixed trophic impact (MTI) routine was applied to quantify both direct and indirect trophic effects among functional groups, indicating how changes in the biomass of one group influence others. (4) The overall ecosystem status was evaluated using global model indices. The connectance index (CI) and system omnivory index (SOI) reflect the complexity of trophic linkages among functional groups, with values closer to 1 indicating more complex food web structures. Finn's cycling index (FCI) and Finn's mean path length (FML) represent the proportion of recycled flows and the average length of energy pathways, respectively, and are commonly used as indicators of ecosystem maturity, with higher values indicating greater maturity. In addition, the ratio of total primary production to total respiration (TPP/TR) is an important indicator of system maturity, with values closer to 1 suggesting more efficient utilization of primary production and a more mature ecosystem (Han et al. 2017; Christensen et al. 2005).

### 2.5 Statistical Analysis

To examine variation in reef‐dwelling animal diversity indices and microbial alpha diversity indices across tidal zones, one‐way analysis of variance (ANOVA) or the non‐parametric Kruskal–Wallis test was used using Origin Pro 2022, depending on whether the data met the assumptions of parametric analysis. Differences were considered statistically significant at the 0.05 level (*p* < 0.05).

## 3 Results

### 3.1 Ecosystem Composition Across Trophic Levels

**FIGURE 2:**
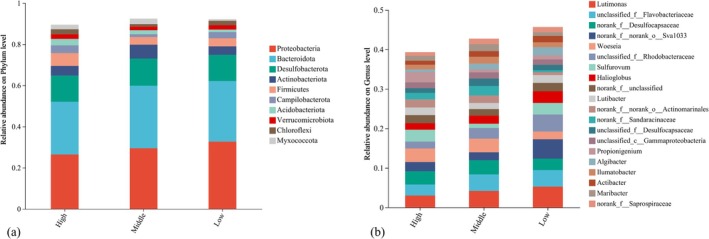
Composition and relative abundance of microbial communities across different tidal zones. (a, b) show the phylum and genus levels, respectively. High, Middle, and Low represent the high‐, middle‐, and low‐tidal zones, respectively (the same below).

**FIGURE 3:**
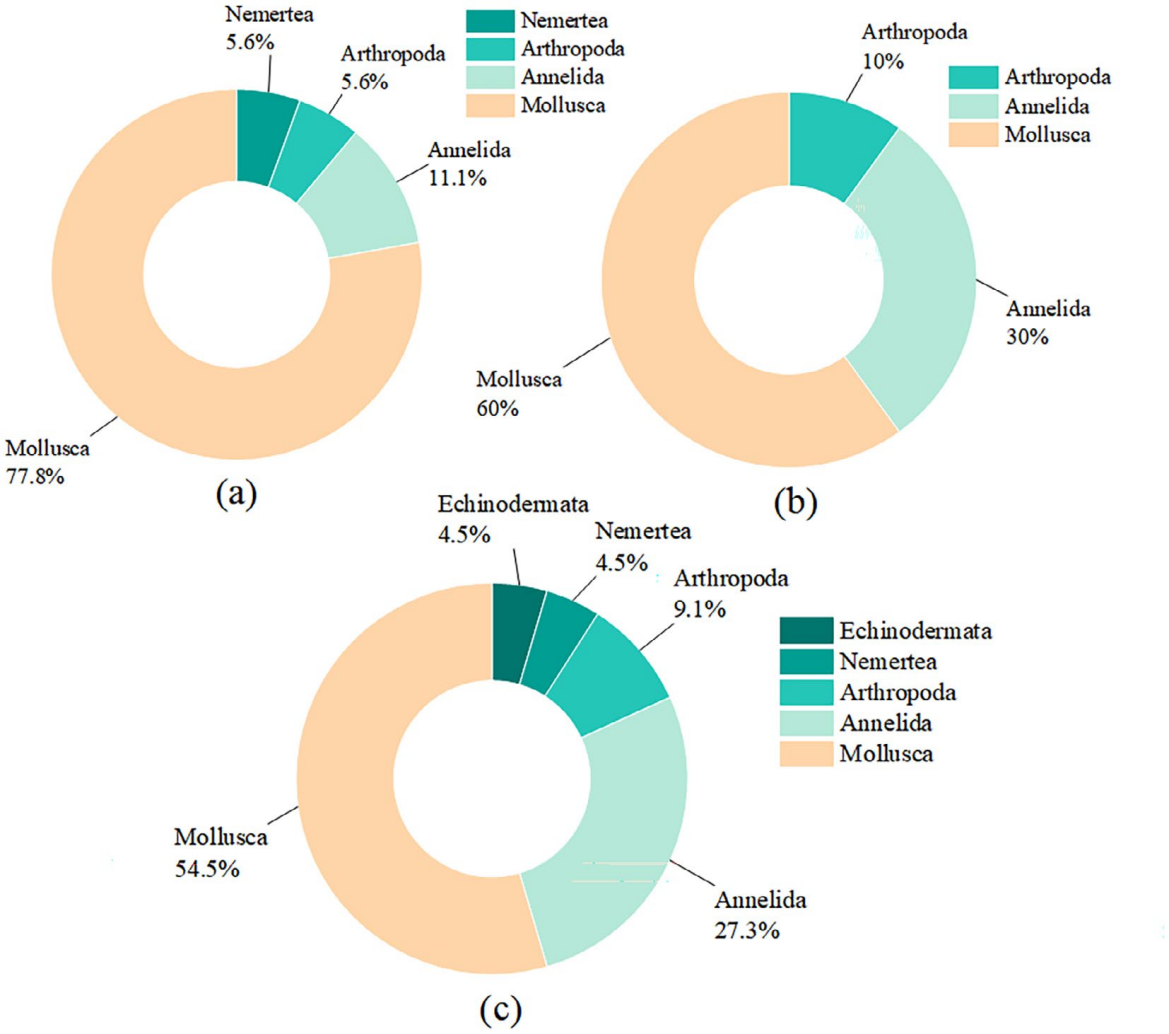
Composition of reef‐dwelling animals across different tidal zones and the proportion of species in each category. (a–c) represent the high‐, middle‐, and low‐tidal zones, respectively, with 18, 20, and 22 species recorded.

**FIGURE 4:**
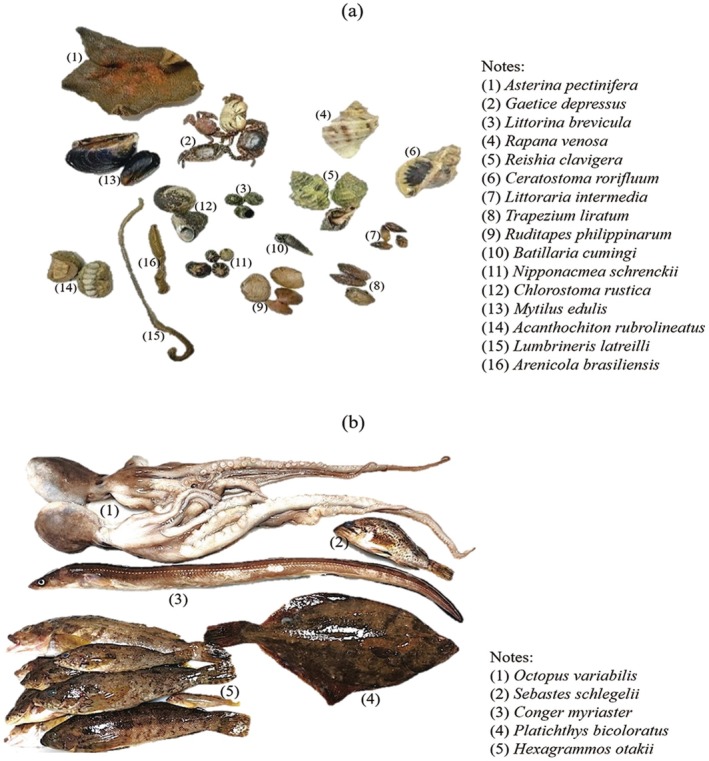
Representative species of reef‐dwelling animals (a) and nekton (b) in *CORE*.

**FIGURE 5:**
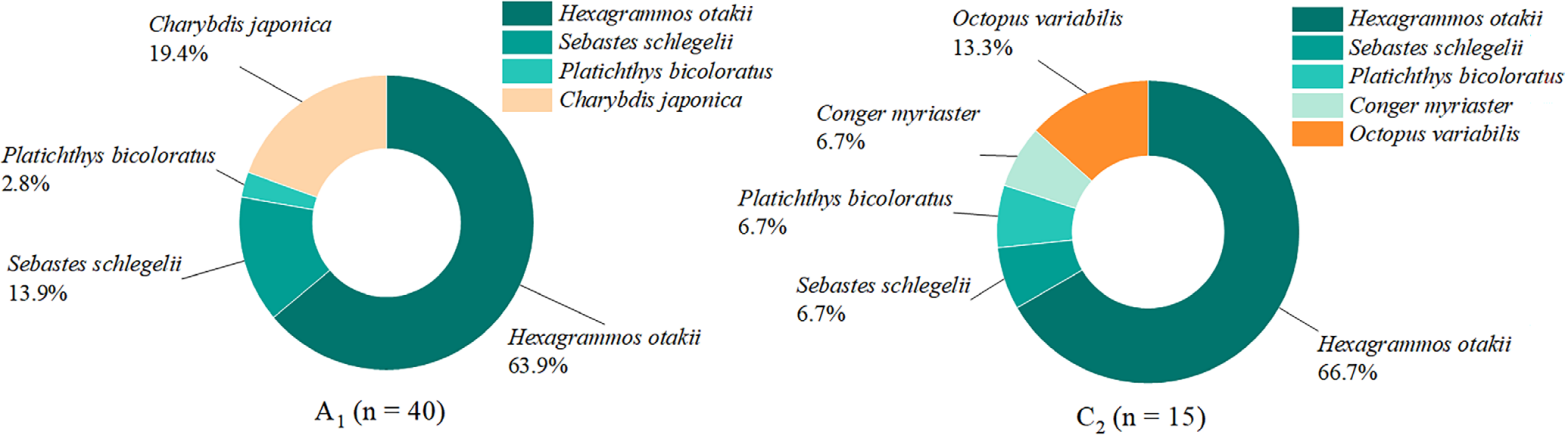
Composition and relative abundance of nekton at different stations in *CORE*.

*CORE* exhibited a multi‐trophic biological structure characterized by oyster dominance and relatively diverse reef‐dwelling animal communities. At the lower trophic levels, phytoplankton assemblages consisted of 13 species, all of which were diatoms. Transect C exhibited the highest phytoplankton abundance, with *Paralia sulcata* accounting for 66.91% of the total abundance. Zooplankton comprised nine species across four groups, with copepods showing the highest species richness (*S* = 4) (Table 2). Microbial communities were primarily dominated at the phylum level by *Proteobacteria* and *Bacteroidota* (each exceeding 15% relative abundance). At the genus level, *Lutimonas* and several unclassified taxa were most abundant, collectively accounting for more than 10% of the total relative abundance (Figure 2). At intermediate trophic levels, oyster density ranged from 1335 to 1605 ind m^−2^, biomass from 12.87 to 14.04 kg m^−2^, and recruitment density (< 20 mm shell height) from 569 to 784 ind m^−2^. Other reef‐dwelling animal density ranged from 297 to 553 ind m^−2^, with biomass between 0.35 and 0.58 kg m^−2^ (Table 3). Table 4 provides a checklist of sedentary reef‐dwelling animals recorded in *CORE*. A total of 30 species belonging to five phyla were identified: Echinodermata, Nemertea, Arthropoda, Annelida, and Mollusca. Based on the proportion of species (Figure 3), mollusks represented the largest group (> 50%) in all three intertidal zones. Oysters from *CORE* were predominantly *Magallana gigas* (formerly *Crassostrea gigas* ), with 26 of 27 individuals genetically identified as this species. One ungenotyped individual was excluded due to insufficient DNA yield, likely caused by poor tissue preservation or extraction conditions. Some representative reef‐dwelling animal species are shown in Figure 4a. At higher trophic levels, a total of six nekton species from three major groups were recorded: *Hexagrammos otakii* , *Sebastes schlegelii* , *Platichthys bicoloratus* , and *Conger myriaster* (Chordata); *Charybdis japonica* (Arthropoda); and *Octopus variabilis* (Mollusca). Among them, *Hexagrammos otakii* had the highest relative abundance at both sites, exceeding 60% at both (Figure 5). Some representative nekton species collected during the survey are shown in Figure 4b.

**TABLE 2:** Species composition and abundance of plankton across transects A, B, and C.

Category	Species	*A*	*B*	*C*	Total	Occurrence
Zooplankton	*Calanus sinicus*	1	3	9	**13**	A, B, C
	*Paracalanus parvus*	2	0	6	**8**	A, C
	*Acartia pacifica*	0	1	1	**2**	B, C
	Cirripedia nauplius larvae	0	1	3	**4**	B, C
	*Oikopleura dioica*	0	4	0	**4**	B
	*Centropages abdominalis*	3	0	0	**3**	A
	*Euphausia diomedeaea* zoea larvae	0	2	0	**2**	B
	*Aidanosagitta crassa*	0	1	0	**1**	B
	*Brachyura* zoea larvae	0	0	1	**1**	C
	**Number of species**	**3**	**6**	**5**	—	—
	**Total abundance**	**6**	**12**	**20**	—	—
Phytoplankton	*Paralia sulcata*	0	0	180	**180**	C
	*Licmophora abbreviata*	24	18	43	**85**	A, B, C
	*Synedra* sp.	54	7	17	**78**	A, B, C
	*Nitzschia* sp.	1	6	12	**19**	A, B, C
	*Nitzschia closterium*	18	0	0	**18**	A
	*Pseudo‐nitzschia pungens*	4	0	5	**9**	A, C
	*Nitzschia lorenziana*	7	0	0	**7**	A
	*Skeletonema costatum*	0	0	5	**5**	C
	*Guinardia delicatula*	0	0	4	**4**	C
	*Coscinodiscus* sp.	0	1	2	**3**	B, C
	*Rhizosolenia setigera*	1	0	0	**1**	A
	*Pleurosigma* sp.	0	1	0	**1**	B
	*Navicula* sp.	0	0	1	**1**	C
	**Number of species**	**7**	**5**	**9**	—	—
	**Total abundance**	**109**	**33**	**269**	—	—

*Note:* Bold values highlight the key results for easier interpretation and comparison.

**TABLE 3:** Oyster and other reef‐dwelling animal community indices (±SD) in different tidal zones.

Group	Index	High‐tide zone	Mid‐tide zone	Low‐tide zone
Oyster	Density (ind m^−2^)	1335 ± 169	1605 ± 560	1367 ± 276
	Recruitment density (ind m^−2^)	569 ± 96	784 ± 438	572 ± 381
	Biomass (kg m^−2^)	13.09 ± 8.39	14.04 ± 4.37	12.87 ± 1.08
Other reef‐dwelling animal	Density (ind m^−2^)	553 ± 334	395 ± 112	297 ± 55
	Biomass (kg m^−2^)	0.58 ± 0.65	0.40 ± 0.08	0.35 ± 0.24

**TABLE 4:** Faunal inventory of reef‐dwelling organisms in Chudao oyster reefs.

Phylum	Species	Phylum	Species
Mollusca	*Magallana gigas*	Echinodermata	*Asterina pectinifera*
	*Patelloida pygmaea*	Nemertea	*Amphiporus punctatulus*
	*Nipponacmea schrenckii*	Arthropoda	*Fistulobalanus albicostatus*
	*Cellana toreuma*		*Gaetice depressus*
	*Littoraria intermedia*		*Photis longicaudata*
	*Chlorostoma rustica*	Annelida	*Marphysa sanguinea*
	*Batillaria cumingi*		*Lumbrineris latreilli*
	*Nassarius variciferus*		*Nereis longior*
	*Rapana venosa*		*Neanthes japonica*
	*Ceratostoma rorifluum*		*Perinereis aibuhiensis*
	*Reishia clavigera*		*Haploscoloplos elongatus*
	*Mytilus edulis*		*Mediomastus californiensis*
	*Trapezium liratum*		*Notomastus aberans*
	*Ruditapes philippinarum*		*Arenicola brasiliensis*
	*Acanthochiton rubrolineatus*		
	*Littorina brevicula*		

### 3.2 Spatial Variation in Biodiversity

**FIGURE 6:**
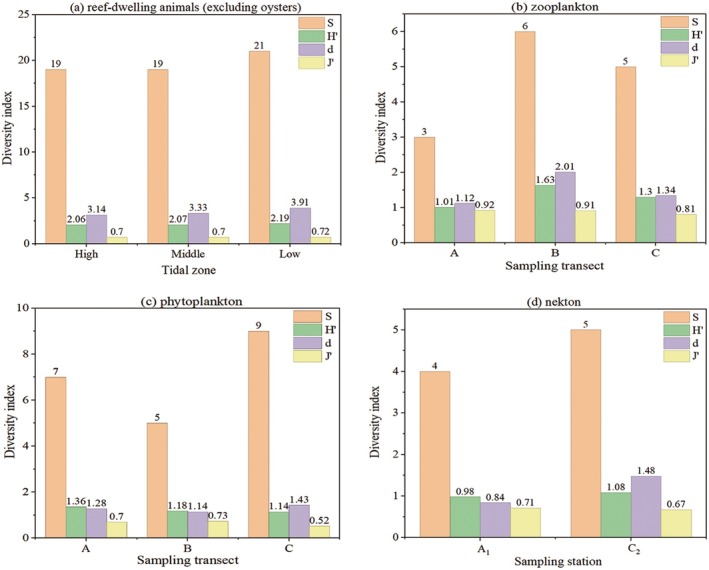
Diversity indices for different biological groups.

Biodiversity in *CORE* showed limited spatial differentiation across tidal zones for most biological components (*p* > 0.05), with a consistent trend of marginally higher diversity in the low‐tide zone. As shown in Figure 6a, the diversity indices of reef‐dwelling animals (excluding oysters) in different tidal zones showed that *S* ranged from 19 to 21, *d* from 3.14 to 3.91, *J*′ from 0.70 to 0.72, and H′ from 2.06 to 2.19. The low‐tide zone had the highest species richness (*d* = 3.91), evenness (*J*′ = 0.72) and diversity (*H*′ = 2.19). One‐way ANOVA showed that *S*, *d*, *J*′, and *H*′ did not differ significantly among tidal zones (*S*: *p* = 0.67; *d*: *p* = 0.29; *J*′: *p* = 0.48; *H*′: *p* = 0.28), with all *p*‐values > 0.05. *Gaetice depressus*, *Nipponacmea schrenckii*, *Mytilus edulis* , *Ruditapes philippinarum*, and *Acanthochiton rubrolineatus* were dominant across all tidal zones. Microbial alpha diversity indices (ACE, Chao, Shannon, and Simpson) were comparable across tidal zones (Table 5), indicating relatively stable diversity patterns. The low‐tide zone exhibited relatively higher richness (ACE, Chao, and Sobs), whereas the high‐tide zone showed relatively higher diversity. One‐way ANOVA showed no significant differences in alpha diversity indices among tidal zones (ACE: *p* = 0.94; Chao: *p* = 0.93; Sobs: *p* = 0.93; Shannon: *p* = 0.98; Simpson: *p* = 0.91; Coverage: *p* = 0.73), with all *p*‐values > 0.05.

**TABLE 5:** Alpha diversity indices (±SD) of microbial communities in different tidal zones.

Index	High‐tide zone	Mid‐tide zone	Low‐tide zone
ACE	1893.49 ± 548.94	1807.53 ± 337.51	1894.34 ± 167.36
Chao	1878.03 ± 533.10	1792.58 ± 333.20	1883.92 ± 175.94
Sobs	1874 ± 528	1787 ± 333	1881 ± 178
Shannon	6.51 ± 0.48	6.44 ± 0.34	6.49 ± 0.35
Simpson	0.0040 ± 0.00	0.0048 ± 0.00	0.0048 ± 0.00
Coverage	0.9983 ± 0.00	0.9981 ± 0.00	0.9988 ± 0.00

For plankton, diversity varied among transects rather than tidal zones (Figure 6b,c). Zooplankton diversity and richness peaked in Transect B (*H*′ = 1.63, *d* = 2.01). For phytoplankton, richness was highest in Transect C (*S* = 9), whereas Shannon diversity and evenness reached their maxima in Transects A (*H*′ = 1.36) and B (*J*′ = 0.73), respectively. Dominant zooplankton species included *Calanus sinicus* , *Paracalanus parvus* , Cirripedia nauplius larvae, *Oikopleura dioica* , *Centropages abdominalis* , and *Euphausia diomedeaea* zoea larvae. Dominant phytoplankton species were the diatoms *Licmophora abbreviata* , *Synedra* sp., *Paralia sulcata* , and *Nitzschia* sp. Nekton diversity differed slightly between stations (Figure 6d). *C*_2_ showed higher species richness (*S* = 5) and Margalef index (*d* = 1.48) than *A*_1_ (*S* = 4, *d* = 0.84), and also had a slightly higher Shannon–Wiener index (*H*′ = 1.08 vs. 0.98). *Hexagrammos otakii* , *Sebastes schlegelii* , *Platichthys bicoloratus* , *Charybdis japonica* , and *Octopus variabilis* were the dominant species at *CORE*.

### 3.3 Trophic Structure and Food Web Organization

**FIGURE 7:**
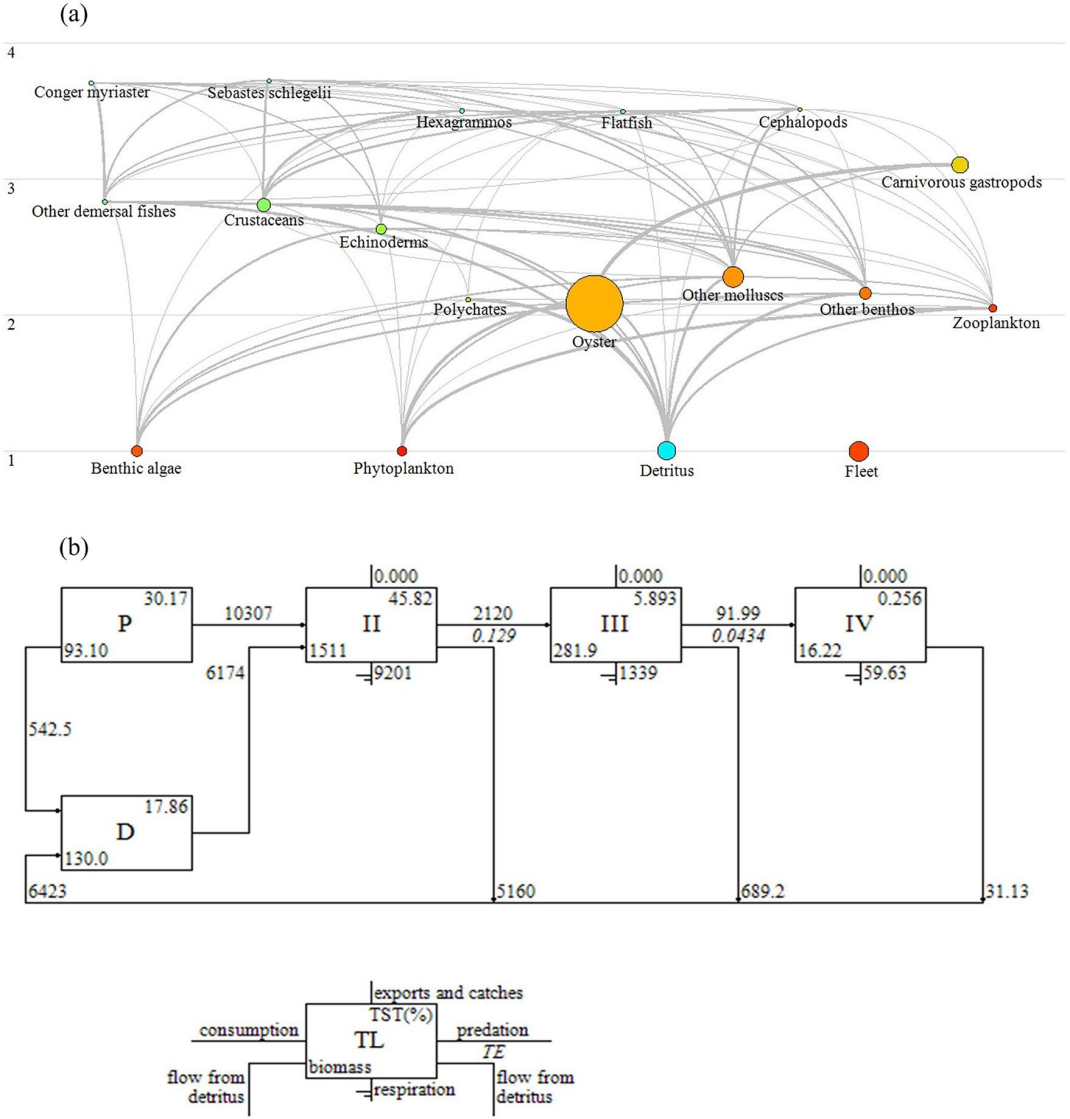
Food web (a) and energy flow diagram (b) of *CORE*.

**FIGURE 8:**
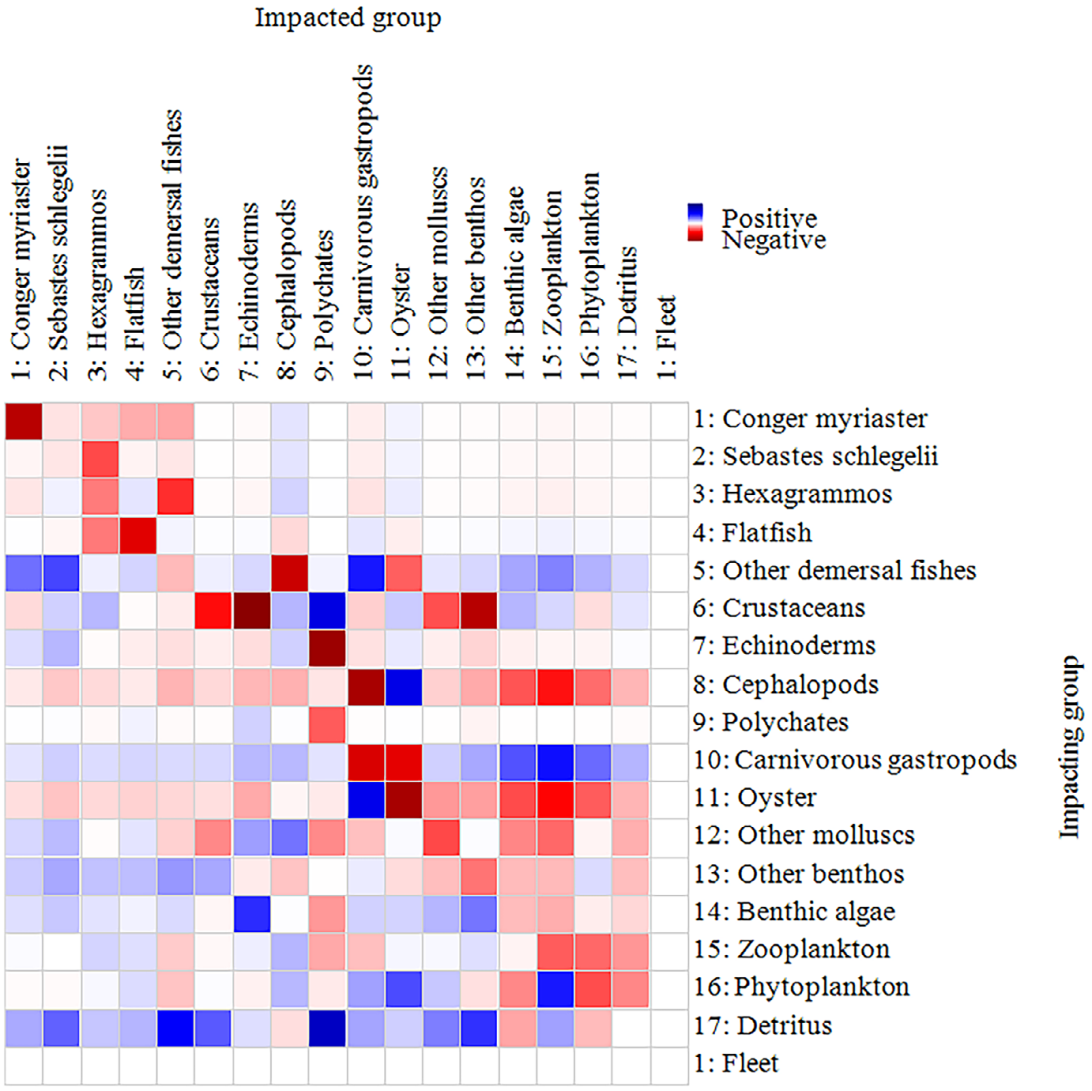
Mixed trophic impact (MTI) analysis of *CORE*.

Ecopath model outputs (Table 6) reveal that the 17 functional groups span a trophic level range of 1.00 to 3.73, forming a food web characterized by structural clarity and relative simplicity. Primary producers and detritus constituted the base of the food web, while oysters and other benthic invertebrates occupied intermediate trophic positions. Higher trophic levels were represented by demersal fishes and cephalopods, with *Sebastes schlegelii* and *Conger myriaster* occupying the highest trophic levels (3.73 and 3.71). In addition, the food web structure (Figure 7a) revealed two main energy pathways: (1) a grazing food chain, in which energy flows from primary producers to benthic invertebrates and subsequently to higher trophic‐level fishes; and (2) a detrital food chain, in which detritus supports benthic consumers and ultimately higher trophic levels. Mixed trophic impact (MTI) analysis indicated strong negative effects of predators on prey and positive bottom‐up effects of prey on predators. Competition among fish groups resulted in mutual negative impacts, while detritus, plankton, and benthic algae generally exerted positive influences on most functional groups. Oysters showed predominantly negative impacts on other groups, reflecting their competitive dominance within the ecosystem (Figure 8).

**TABLE 6:** Input and output parameters in the Ecopath model of *CORE*.

Functional group	TL	Biomass (t km^−2^)	P/B (year^−1^)	Q/B (year^−1^)	EE	P/Q (year^−1^)
*Conger myriaster*	3.71	0.2785	0.90	4.80	0.107	0.188
*Sebastes schlegelii*	3.73	0.1175	0.90	6.80	0.000	0.132
*Hexagrammos*	3.50	1.3290	0.70	3.40	0.313	0.206
Flatfish	3.50	0.2610	1.12	3.80	0.148	0.295
Other demersal fishes	2.83	1.3178	1.30	9.30	0.950	0.140
Crustaceans	2.81	77.36	5.60	16.90	0.821	0.331
Echinoderms	2.64	42.72	1.30	3.70	0.482	0.351
Cephalopods	3.52	0.16	2.90	12.00	0.950	0.242
Polychaetes	2.11	3.80	6.75	20.60	0.610	0.328
Carnivorous gastropods	3.11	105.08	0.26	2.82	0.007	0.092
Oyster	2.08	1333	1.23	7.75	0.159	0.159
Other mollusks	2.28	161.29	4.40	17.20	0.594	0.256
Other benthos	2.16	59.61	6.40	27.80	0.769	0.230
Benthic algae	1.00	54.49	50.55		0.950	
Zooplankton	2.05	23.38	73.00	122.10	0.950	0.598
Phytoplankton	1.00	38.60	209.70		0.950	
Detritus	1.00	130.00			0.961	

### 3.4 Energy Flow and Ecosystem Functioning

Energy flow analysis revealed that *CORE* is characterized by active energy throughput but relatively low transfer efficiency. Total system throughput (TST) reached 36,752.52 t km^−2^ year^−1^, with primary producers contributing approximately 61% of total energy input. Energy flow was largely concentrated in lower trophic levels (Figure 7b), with trophic level II accounting for the largest proportion (45.82%), followed by trophic level III (5.89%) and IV (0.26%). Energy transfer efficiency between trophic levels was generally low, with an overall efficiency of 4.21%, substantially lower than the classical Lindeman efficiency of 10%. Transfer efficiency was highest between trophic levels II and III, indicating relatively efficient energy transfer at intermediate trophic levels (Table 7). Ecosystem‐level indicators suggested that *CORE* is relatively mature and stable. The CI and SOI were 0.37 and 0.22, indicating a complex food web and relatively high system stability. The TPP/TR was 1.02, suggesting efficient utilization of primary production and a relatively mature ecosystem. Additionally, the FCI (14.69) and FML (3.39) were also higher than in other ecosystems, further indicating high ecosystem maturity (Table 8).

**TABLE 7:** Transfer efficiency (%) of discrete trophic levels in *CORE*.

Source/Trophic level	II	III	IV
Producer	12.55	4.48	1.27
Detritus	13.39	4.12	1.46
All flows	12.86	4.34	1.34
Proportion of total flow originating from detritus: 0.37			
From primary producers: 4.15%			
From detritus: 4.31%			
Total: 4.21%			

**TABLE 8:** Comparison of ecosystem attributes of the *CORE* model with other models.

Attribute parameter	This paper (*CORE*)	Jiaozhou Bay, China (Han et al. 2017)	Lidao artificial reef, China (Wu et al. 2016)	Artificial Reef in Laizhou Bay, China (Yuan et al. 2022)	Galapagos subtidal rocky reef, Ecuador (Okey 2004)	Red Sea coast Eritrean (Tsehaye and Nagelkerke 2008)	Rocky coastal ecosystem Bahia Tortugas, Mexico (Morales‐Zárate et al. 2011)	Subtidal area in Tongoy Bay Chile (Ortiz and Wolff 2002)
Total system throughput (TST) (t km^−2^ year^−1^)	36,752.52	12,917.10	11,104.00	17,300.57	94,850.00	66,249.00	553.00	20,594.00
Calculated total net primary production (TPP) (t km^−2^ year^−1^)	10,849.86	5626.22	1865.20	1041.12	13,250.00	18,179.00	—	8541.60
Total primary production/total respiration (TPP/TR)	1.02	3.18	1.82	1.21	0.48	1.00	1.05	2.70
Connectance Index (CI)	0.37	0.31	0.32	0.21	0.16	0.46	0.23	0.20
System Omnivory Index (SOI)	0.22	0.16	0.14	0.09	0.25	0.21	0.23	0.14
Finn's cycling index (FCI)	14.69	2.47	5.46	76.15	—	10.76	—	2.60
Finn's mean path length (FML)	3.39	2.30	2.69	16.62	—	3.64	—	2.41

Sensitivity analysis showed that introducing a low fishing mortality rate had negligible effects on ecosystem characteristics. Key indicators (including trophic transfer efficiency, TST, and FCI) varied by less than 5% compared with the baseline model. These results indicate that model outputs were insensitive to small changes in fishing mortality, and the *F* = 0 assumption did not significantly affect energy flow patterns or ecosystem structure in *CORE*.

## 4 Discussion

### 4.1 Tidal Zone Characteristics in Oyster Reef Ecosystems

Tidal zonation is a fundamental structuring factor for intertidal ecosystems, as aerial exposure duration drives gradients in desiccation stress, temperature, and oxygen availability, which typically shape vertical distribution patterns of benthic organisms (Inaba and Hall‐Spencer 2020; Eston et al. 1986). However, the present study found no significant biodiversity differences across tidal zones in *CORE*. This homogeneity may be attributed to two key factors: First, the small spatial scale and mild environmental gradients of *CORE* (oyster reef area ≈ 0.29 km^2^) result in negligible differences in abiotic factors (temperature, salinity, DO, SPM) across tidal zones (Table 1), reducing environmental filtering on benthic communities. Second, reef‐associated organisms may have a high adaptive capacity to tidal stress. Dominant species (e.g., *Magallana gigas*, *Gaetice depressus*, *Nipponacmea schrenckii*) are typical intertidal species with robust physiological adaptations to short‐term aerial exposure, minimizing interspecific competition along tidal gradients. The marginally higher diversity in the low‐tide zone is consistent with the intermediate disturbance hypothesis, as low‐tide zones have longer immersion time, higher food availability (e.g., phytoplankton, detritus), and milder environmental stress, supporting more species.

It is important to note that tidal zonation was not incorporated into the sampling design for nekton and plankton. Reef‐associated organisms are generally sessile or weakly motile and are therefore strongly affected by tidal exposure, making tidal‐zone‐based sampling appropriate for capturing their spatial distribution patterns. In contrast, plankton and nekton are more mobile and are influenced primarily by hydrodynamic processes rather than by tidal elevation per se. Therefore, transect‐based sampling was considered sufficient to characterize their community structure. Nevertheless, future studies could incorporate tidal‐zone‐based sampling for these groups to further improve understanding of spatial variability.

A caveat of this study is that sampling was conducted during a single season (May 2024). In temperate coastal systems, spring is typically associated with high primary productivity and intense biological activity (Hjerne et al. 2019). As sampling was restricted to this season, the results may not fully capture seasonal biodiversity patterns across tidal zones. Seasonal variation in temperature, recruitment, and hydrodynamic conditions is likely to influence species distribution and diversity in *CORE*. For example, winter cold stress may increase mortality in the high‐tide zone, potentially resulting in more pronounced diversity differences among tidal zones. Therefore, multi‐seasonal investigations are needed to determine whether the spatial patterns observed in this study persist over time.

### 4.2 Ecological Characteristics of *CORE*

The Ecopath model indicates that *CORE* is characterized by active energy flow and a relatively well‐developed trophic structure. The relatively high total system throughput (TST = 36,752.52 t km^−2^ year^−1^) suggests that the ecosystem maintains a high level of activity compared with many other coastal ecosystems (Table 8). Energy flow was concentrated predominantly at lower trophic levels, especially trophic level II, highlighting the key role of benthic suspension feeders, particularly oysters, in ecosystem energy transfer. As suspension‐feeding bivalves, oysters serve as key intermediaries linking primary producers, suspended organic matter, and higher trophic levels. By efficiently filtering phytoplankton and particulate organic matter, oysters transform primary production into benthic biomass that is subsequently available to predators. This trophic configuration is typical of coastal ecosystems dominated by bivalves, where filter feeders enhance benthic–pelagic coupling and facilitate the transfer of energy from lower to higher trophic levels (Newell et al. 2005; Dame 1993). In *CORE*, the high biomass of oysters further strengthens this pathway, resulting in a food web that is strongly structured by benthic processes.

The food web of *CORE* was sustained by both grazing and detrital pathways, indicating close coupling among primary production, benthic consumers, and detrital recycling. This dual‐pathway structure is characteristic of productive coastal ecosystems, where detrital pathways provide an additional energy source, buffer fluctuations in primary production, and contribute to food‐web stability (Colléter et al. 2015). In *CORE*, the relatively high FCI and FML values suggest strong internal recycling, while the TPP/TR value close to 1 indicates a comparatively mature and self‐sustaining ecosystem. This distinguishes *CORE* from many coastal oyster reef systems that remain more strongly production‐dominated. The relatively mature state of *CORE* is likely associated with the habitat mosaic of the Chudao marine ranching area, where oyster reefs, seaweed aquaculture, and artificial reefs together increase habitat heterogeneity, support diverse trophic groups, and potentially strengthen trophic interactions (Zhang et al. 2022; Christensen et al. 2005).

Although the overall trophic transfer efficiency of *CORE* was low, this pattern is consistent with the ecological characteristics of benthic filter‐feeder‐dominated systems. Several mechanisms may account for this low efficiency. First, substantial energy is lost during oyster filter‐feeding, as a large proportion of ingested particulate organic matter is not converted into biomass but is instead released as feces and pseudofeces and subsequently redirected into the detrital pool. Second, respiratory losses at lower trophic levels are considerable, particularly among zooplankton and benthic invertebrates with relatively high P/B and Q/B ratios. Third, compared with the grazing pathway, the detrital pathway is generally less efficient in transferring energy to higher trophic levels because decomposition and transformation involve multiple intermediate steps and cumulative energy losses (Hurst et al. 2022). Nevertheless, the relatively high transfer efficiency between trophic levels II and III suggests that intermediate consumers, such as crustaceans and echinoderms, still play an important role in channeling oyster reef‐derived energy to higher trophic levels and maintaining trophic connectivity. Therefore, the ecological functioning and long‐term stability of *CORE* depend not only on oysters as the structural and trophic foundation of the ecosystem but also on the persistence of intermediate consumers that link benthic production to higher trophic levels. Taken together, *CORE* can be characterized as a mature, oyster‐dominated reef ecosystem with ongoing expansion.

### 4.3 Functional Group Classification and Model Uncertainty

Functional group classification is a key step in Ecopath modeling because it determines how trophic interactions and energy pathways are represented. In the present study, organisms in *CORE* were classified into 17 functional groups according to ecological roles, feeding habits, habitat characteristics, and data availability. Fish species were treated as separate groups because of their relatively high trophic positions and distinct roles in trophic regulation, whereas several benthic invertebrates were aggregated into broader categories to maintain a balance between ecological realism and model parsimony. At the same time, oysters and carnivorous gastropods were retained as independent groups because of their prominent functional importance in the reef ecosystem, particularly in benthic energy transfer and predator–prey interactions (Wang et al. 2023; Astudillo et al. 2018; Hu et al. 2016). Although this grouping strategy follows common practice in Ecopath studies, some uncertainty is unavoidable. In particular, several input parameters, including P/B, Q/B, and diet composition, were derived from published studies on adjacent or comparable ecosystems rather than from direct local measurements.

A limitation of the present Ecopath model is that microorganisms were not explicitly represented as a separate functional group. Given their involvement in organic matter decomposition, nutrient regeneration, and other detritus‐related biogeochemical processes in oyster reef ecosystems (Green et al. 2012), this omission may have led to a simplified representation of microbial recycling and internal nutrient turnover. Consequently, the model may be less effective in resolving fine‐scale detrital transformations. Nevertheless, this limitation is unlikely to substantially affect the interpretation of broader trophic structure and system‐level energy flow patterns. Thus, the main findings regarding benthic dominance, the coupling of grazing and detrital pathways, and the relative stability of energy organization in *CORE* remain supported by the model results.

Future studies should incorporate multi‐seasonal observations to capture temporal variability in biodiversity and ecosystem functioning. In addition, integrating microbial functional groups into Ecopath models would improve the representation of detrital pathways and internal nutrient cycling. Coupling trophic models with hydrodynamic and biogeochemical measurements may further enhance our understanding of ecosystem connectivity between oyster reefs and adjacent habitats, and help clarify their broader ecological role in this coastal region.

## 5 Conclusion

This study quantitatively evaluated the ecosystem structure and functioning of *CORE* based on integrated whole‐biota observations and an Ecopath model. A total of 30 reef‐dwelling animal species were identified, with oysters dominating both density and biomass. Biodiversity showed limited spatial variability across tidal zones, with slightly higher diversity in the low‐tide zone. The Ecopath model included 17 functional groups with trophic levels ranging from 1.00 to 3.73. Total system throughput reached 36,752.52 t km^−2^ year^−1^, and primary producers contributed approximately 61% of the total energy input. Ecosystem indicators (CI = 0.37, SOI = 0.22, FCI = 14.69, TPP/TR = 1.02) indicate that *CORE* is a relatively mature and stable ecosystem. However, the low energy transfer efficiency (4.21%) suggests substantial energy dissipation during trophic transfer, reflecting the inherent characteristics of coastal detritus‐based food webs. These findings highlight the fundamental role of healthy oyster reefs in supporting coastal biodiversity, regulating energy flow, and maintaining food web stability. As the first comprehensive ecosystem‐level assessment of this rare expanding oyster reef, the present study establishes a critical baseline for future studies on ecosystem connectivity and adaptive management.

## Author Contributions

**Yazhou Shi:** writing – original draft (lead). **Qisheng Tang:** writing – review and editing (equal). **Yaping Gao:** writing – review and editing (supporting). **Ruihuan Li:** writing – review and editing (supporting). **Weiwei Li:** investigation (equal). **Mingjun Yuan:** investigation (equal). **Linjie Wang:** investigation (equal). **Shujie Chang:** investigation (equal). **Zengjie Jiang:** writing – review and editing (lead).

## Funding

This work was supported by State Key Laboratory of Mariculture Biobreeding and Sustainable Goods Basal Research Fund (BRESG‐JB202412), Central Public‐interest Scientific Institution Basal Research Fund, Chinese Academy of Fishery Sciences (2023TD54), Key Programme for International Cooperation on Scientific and Technological Innovation, Ministry of Science and Technology (2025YFE0109400), National Natural Science Foundation of China, 42376151, the earmarked fund for CARS (CARS‐49).

## Conflicts of Interest

The authors declare no conflicts of interest.

## Data Availability

The original data supporting this study are available in the following digital repository: https://github.com/shiyazhou331/Repository‐name1.git.
